# Milk kefir drink may not reduce depression in patients with non-alcoholic fatty liver disease: secondary outcome analysis of a randomized, single-blinded, controlled clinical trial

**DOI:** 10.1186/s40795-023-00732-x

**Published:** 2023-06-29

**Authors:** Mohammad Ali Mohsenpour, Farzaneh Mohammadi, Nadia Razmjooei, Mohammad Hassan Eftekhari, Najmeh Hejazi

**Affiliations:** 1 Student Research Committee, Shiraz University of Medical Sciences, Shiraz, Iran; 2 Department of Clinical Nutrition, School of Nutrition and Food Sciences, Shiraz University of Medical Sciences, Shiraz, Iran

**Keywords:** Non-alcoholic fatty liver disease, Depression, Depressive disorder, Milk, Kefir

## Abstract

**Background:**

Depression is prevalent among individuals with non-alcoholic fatty liver disease (NAFLD) and can cause poor health outcomes. Moreover, a solid bilateral association between NAFLD and depression has been shown, which may alleviate by kefir consumption. Thus, we aimed to investigate the effect of milk kefir drinks on the depression status of individuals with NAFLD.

**Methods:**

In a secondary outcome analysis of a randomized, single-blinded, controlled clinical trial, 80 adults with grades 1 to 3 of NAFLD were included in an 8-week intervention. Participants were randomly assigned to Diet or Diet + kefir groups to either follow a low-calorie diet or a low-calorie diet along with a 500 cc milk kefir drink daily. The participants’ demographic, anthropometric, dietary, and physical data were recorded before and after the study. Depression status was assessed using the Persian format of the second version of the Beck Depression Inventory (BDI-II-Persian) at the baseline and after 8 weeks of intervention.

**Results:**

Overall, 80 participants aged 42.87 ± 10.67 years were included in the analysis. The data on the baseline demographic, dietary, and physical activity of the groups were not significantly different. During the study, participants in Diet + Kefir group had a significantly decreased energy (P = 0.02), carbohydrate (P = 0.4), and fat consumption (P = 0.4). However, during the study, the depression score was not significantly reduced in the Diet group, the Diet + Kefir group showed a significant reduction in depression (P = 0.02). However, between-group analyses for changes in depression were not significant (P = 0.59).

**Conclusion:**

Consumption of milk kefir drink for 8 weeks may not reduce depression symptoms in adults with NAFLD.

**Trial registration:**

The trial was registered at IRCT.ir as IRCT20170916036204N6 (August 2018).

## Background

Non-alcoholic fatty liver disease (NAFLD) is caused by over-accumulation of fat in the liver [1]. Progression of NAFLD can increase the odds of cardiovascular diseases, type 2 diabetes mellitus (T2DM), extra-hepatic cancer, and can eventually lead to higher mortality and morbidity [1].

The co-occurrence between depression and physical illnesses has been established [2]. NAFLD and depression are shown to have a solid bilateral association [3]. It was observed that depression is highly prevalent among NAFLD patients in which 18.21% of patients suffer from depression [3]. In addition, the 10-year incidence rate of depression was 21.2% in NAFLD patients in comparison to 18.2% in non-NAFLD individuals [4].

Depression is one of the mental disorders that is considered one of the serious concerns in today’s modern society [5]. It is predicted to be the second cause of the burden of disease by 2030 [5]. Depression can lead to suicide, increased mortality risk, and chronic status, and lower occupational potential and the quality of life of the depressed individual [6]. This mental disorder affects 12.9% of individuals globally, where the prevalence differs based on the region, human development index of countries, gender, and other socioeconomic factors [6]. In Asian countries, 16.7% of the population suffer from depression [6]. In 2019, the total prevalence of depression in Iran was 4.1% [7].

It is stated that depression inversely affects health outcomes and the progression of the disease [8, 9]. Based on the findings of community-based studies, metabolic syndrome incidence is 2 times higher in individuals with depression [10, 11]. Conducting a meta-analysis of longitudinal studies has resulted in a reciprocal causal relationship between depression and abnormal weight [12]. Also, NAFLD patients with depression showed a lower rate of improvement [13] and higher odds of 1-year mortality compared to non-depressed patients [14]. Thus, the central role of depression in multimorbidity and the increase in the incidence of chronic diseases justify the screening and intervention programs for those who suffer and are at risk of chronic diseases [15].

Several dietary interventions have been investigated to reduce depressive symptoms including adherence to the Mediterranean diet [16], the Mediterranean-DASH Intervention for Neurodegenerative Delay (MIND) diet [17], Dietary Approaches to Stop Hypertension (DASH) diet [18], milk [19], and kefir consumption [20].

Kefir is a fermented dairy beverage generated after the lactose content of milk is fermented by bacteria and yeasts [21]. Kefir drink contains various favorable compounds such as essential amino acids, vitamins, folic acid, bioactive substances, and minerals [22], which exert health promoting effects such as anti-cancer, anti-diabetic, anti-hypertensive, anti-inflammatory, and hypo-cholesterolemic property [21]. In addition, depression was seen to be prevented and treated by consumption of kefir [20], which is proposed to act through gut microbiota [23] and neural pathways [24].

Although pieces of evidence have shown the causal relationship between NAFLD and depression, the adverse effects of depression in the treatment of chronic diseases, and the favorable effects of milk and kefir in improving depression symptoms, no studies have been conducted to assess the effect of kefir on depressive disorders in NAFLD patients. Thus, this study aimed to investigate the effect of milk kefir drink on depression status in individuals with NAFLD.

## Methods

### Study design

The present study is a secondary outcome analysis of a randomized, single-blinded, controlled clinical trial to investigate the effect of milk kefir drink on the depression status of adults with NAFLD. The study was done in Motahari Fatty Liver Clinic affiliated with Shiraz University of Medical Sciences in accordance with the Helsinki Declarations of ethics and approved by the Ethics committee of Shiraz University of Medical Sciences (SUMS), Shiraz, Iran (Code: IR.SUMS.REC.1397.107). The study protocol was registered in the Iranian Registry of Clinical Trials (IRCT.ir) under the registration code: IRCT20170916036204N6. The study reporting adheres to the CONSORT guidelines for reporting clinical trials.

### Study population

The study population was obtained from the original study [25]. The original study sample size was calculated 40 based on the changes for aspartate transaminase (AST) in a previous study [26].

Consented adults to participate aged 18 to 65 years old were eligible if they: (1) suffered from grades 1 to 3 of NAFLD based on the confirmation by physicians in regard to sonography results, (2) had elevated alanine transaminase (ALT) (over 30 IU/L for male and 19 IU/L for female participants), (3) were overweight or obese (Body Mass Index (BMI) ≥ 25 kg/m^2^), (4) were under control for Type 2 Diabetes Mellitus (T2DM) or dyslipidemia (if available), (5) were not suffering from other hepatic disorders including cirrhosis, Wilson’s disease, hepatitis B or C, (6) were not diagnosed with cardiovascular, renal, autoimmune, inflammatory bowel diseases, cystic fibrosis, hypothyroidism, or Alpha-1 Antitrypsin Deficiency, (7) did not consume any nutritional supplements or probiotics 3 months prior to the study, (8) did not lose weight for more than 3 kg in 3 months before the study, (9) did not participate in any interventional study for 6 months before the initiation of the present investigation, and (10) were not pregnant or lactating. After inclusion in the study, any participant who used nutritional supplements or changed the dosage of anti-hyperglycemic or anti-hyperlipidemia medications, or did not adhere to the prescribed diet, recommendations, or study protocol was excluded from the study. The threshold of consuming 85% of milk kefir drinks was considered as the least adherence to the study protocol. Moreover, in the case of unwillingness to continue cooperation in the research, the participants were free to leave the study and it did not affect following the common NAFLD medical care.

### Study protocol

After the referral from the physician, the participants were assessed for eligibility. In the case of eligibility, each participant was informed about the study, its aims and protocol, and their own rights; then, an informed consent form was signed by those who were willing to participate in the study. Subsequently, according to the randomization sequence that was generated using computer based on the block randomization method (blocks of 4 with 2:2 ratio) prior to the study, the participants were allocated to either the Diet + Kefir or Diet groups. The groups were named A or B to blind the participants and researchers. Opaque closed envelopes were used to conceal the participants’ allocated group until the initiation of the study period.

After the allocation was completed, the participants entered into a 2-week run-in period to unify their nutritional behavior and remove its confounding effect. For this purpose, a pamphlet was given to them containing general nutritional information for NAFLD patients. Participants were also asked not to change their usual physical activity.

At the end of the run-in period, the participants attended the Fatty liver Clinic. The demographic, nutritional, anthropometric, and physical activity data and the depression status of the participants were assessed and recorded. Afterward, the allocated group for each participant was revealed to the investigator to prescribe the intervention.

The participants were asked to follow the study protocol and the prescribed intervention for 8 weeks. They were also asked to attend the Fatty liver Clinic following completing the eight-week study period to assess their nutritional, physical activity, anthropometry, and depression status.

### Intervention

A low-calorie diet was designed for the participants in face-to-face visits for both groups of Diet + Kefir and Diet. The Estimated Energy Requirement formula was used to calculate the daily energy [27]. A 500-calorie was reduced to design the low-calorie diet. Then, a diet with 55% carbohydrate, 17% protein, and 28% fat was prescribed. Participants in the Diet + Kefir group also consumed 500 cc milk kefir drink daily for 8 weeks (Fars Pegah Dairy Co., Shiraz, Iran), so that these dairy products were a part of their daily diet as 2 dairy servings per day. The energy, carbohydrate, fat, and protein content of milk kefir drink was 118 kcal, 10, 5, and 8 g, respectively, and 300 mg calcium per serving (250 cc). Participants brought empty containers of their milk kefir drink every 2 weeks to determine their adherence and took their quota for the next 2 weeks.

### Demographic assessments

The demographic assessment was done using a questionnaire that included data about age, gender, disease and medication history, food allergies, and smoking.

### Anthropometric assessments

Weight was assessed with the minimum possible clothing using the Seca scale (Germany) with 100-gram accuracy; the participant stood straight in the middle of the scale. Height was recorded using a measuring tape attached to the wall with a barefoot, no hat or scarf, in a standing position, while heels, buttocks, and shoulders touched the wall, and the head was in the Frankfurt position. The height was recorded to the nearest 0.5 cm. BMI was calculated using the standard formula [27]. Waist circumference was assessed by an inelastic tape measure with an accuracy of 0.1 cm in the standing position. It was measured at the end of a usual expiration at the narrowest circumference between iliac crest and the lowest rib. Participants were had the lightest possible clothing, and the tape did not put any pressure on the skin.

### Dietary assessment

A 3-day food recall (2 weekdays and one weekend) was used to record the dietary intake of the participants before and after the study. Food recalls were converted to grams using common household scales of Iranians for each food item and analyzed using Nutritionist 4 software (N4, First Databank Inc., San Bruno, CA, USA). The daily energy, protein, carbohydrate, fat, micronutrient, and fiber consumption were extracted.

### Physical activity

The International Physical Activity Questionnaire (IPAQ) was used to assess the physical activity habits of the participants. This 7-item questionnaire asks about the intensity and duration of physical activity during the past 7 days. The duration of each activity was multiplied by the metabolic equivalent (3.3, 4, and 8 for light, moderate and intense, respectively) to calculate the physical activity of the participants (MET.min/week) [28].

### Depression assessment

The Persian form of the Beck Depression Inventory-II (BDI-II-Persian) was used to assess the depression status of the participants. The BDI-II-Persian asks about depression symptoms’ severity over the past 2-weeks using 21 questions. The BDI-II-Persian includes multiple choice questions with four answers ranging from absent or mild to severe which scored from 0 to 3. The overall score is calculated by summing up the scores (0 to 63). This Persian version of the questionnaire has been checked for validity in the Iranian population [29]. Based on their results in BDI-II-Persian, individuals are categorized as not- or minimally depressed (scored less than 10), mildly depressed (scored 10 to 18), moderately depressed (scored 19 to 29), and severely depressed (scored 30 or higher).

### Statistical analysis

The normal distribution of data was assessed using the Kolmogorov-Smirnov test. Categorical and quantitative data were summarized as frequency (percent) and mean ± standard deviation (SD), respectively. Between-group analyses were done using an independent sample t-test. Paired sample t-test was conducted for within-group comparison. Analysis of covariance (ANCOVA) was carried out to adjust the effect of covariates. Energy, fat, and carbohydrates were considered as covariates in ANCOVA test for adjustments. For dropout participants, the last observation carried forward method was used for imputing the missing data. Statistical analysis was done by SPSS software version 19 (SPSS Inc., Chicago, IL, USA). A P-value less than 0.05 was considered significant.

## Results

**Fig. 1:**
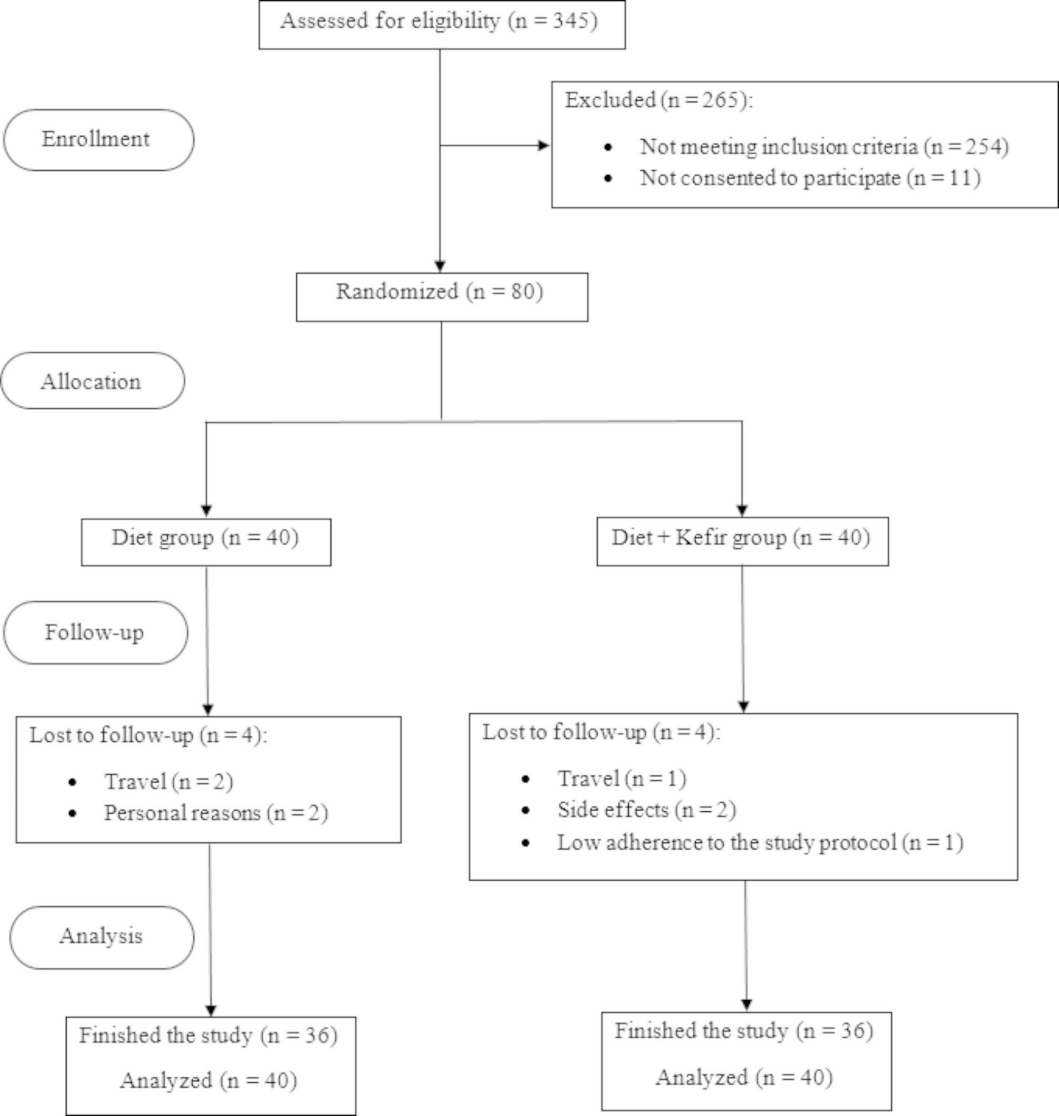
Consort flow diagram of the study procedure

Of 345 individuals assessed for eligibility, 254 were not eligible and 11 did not consent to participate. Eighty adults with NAFLD were allocated to either Diet + Kefir or Diet group. In each Diet + Kefir and Diet group, 4 participants did not finish the study (3 because of travel, 2 participants showed side effects (gastric cramps and bloating), 2 stated personal reasons for leaving the study, and 1 did not adhere to the study protocol). Thus, in each group, 36 subjects successfully completed the study. Figure 1 shows the consort flow diagram for the study procedure.

Table 1 summarizes the demographic characteristics of the participants. Overall, participants enrolled in the study with a mean age of 42.87 ± 10.67 in males and females and 43.8% (n = 35) were male.

**Table 1:** Demographic characteristics of the participants (n = 80)

	Diet (n = 40)	Diet + Kefir (n = 40)	P-value
Age year (mean ± SD)	43.50 ± 11.00	42.25 ± 10.44	0.52^#^
Gender			0.07^†^
Male	13 (32.5)	22 (55.0)	
Female	27 (67.5)	18 (45.0)	
Marital Status			0.57^†^
Single	6 (15.0)	5 (12.5)	
Married	33 (82.5)	32 (80.0)	
Widowed	1 (2.5)	3 (7.5)	
Smoking			0.17^†^
Yes	7 (17.5)	3 (7.5)	
No	33 (82.5)	37 (92.5)	
Medication history			0.36^†^
Yes	18 (45.0)	14 (35.0)	
No	22 (55.0)	26 (65.0)	
Disease history			0.25^†^
Yes	27 (67.5)	22 (55.0)	
No	13 (35.2)	18 (45.0)	

* Data are reported as n (%), otherwise, it is stated

^#^ Independent Sample t-test

^†^ Chi-square test

P-value less than 0.05 considered significant

Table 2 shows the dietary intake, physical activity, and anthropometric characteristics of participants. Baseline results of the dietary intake and physical activity showed no significant differences between the groups (P < 0.05). Although between-group analysis for dietary intake and physical activity at the 8th week did not show any statistically significant differences, the mean changes for energy (P = 0.02), carbohydrate (P = 0.04), and fat (P = 0.04) consumption during the study period showed a higher decrease in the Diet + Kefir group. Anthropometric measures of the participants were not different between the groups at baseline and 8th week (P > 0.05), but after the 8 weeks of intervention, within-group comparison indicated a significant difference in weight (P = 0.01 and P = 0.02 for Diet and Diet + Kefir, respectively), BMI (P = 0.01 for both groups), and waist circumference (P = 0.01 for both groups).

**Table 2:** Dietary intake, physical activity, and anthropometric indices of the participants (n = 80)

		Diet (n = 40)	Diet + Kefir (n = 40)	P-value
Energy (kcal/day)	Baseline	2050.52 ± 552.41	2182.80 ± 561.49	0.29*
	8th week	1938.29 ± 510.84	1815.42 ± 418.58	0.24*
	P-value	0.18^#^	< 0.01^#^	
	Mean differences	− 112.22 ± 521.57	− 367.37 ± 461.63	0.02*
Protein (g/day)	Baseline	70.01 ± 19.24	71.04 ± 25.36	0.83*
	8th week	63.02 ± 17.47	60.37 ± 21.56	0.54*
	P-value	0.03^#^	< 0.01^#^	
	Mean differences	− 6.98 ± 19.62	− 10.67 ± 20.75	0.41*
Carbohydrate (g/day)	Baseline	301.97 ± 93.99	314.29 ± 88.43	0.54*
	8th week	286.95 ± 92.09	246.33 ± 73.42	0.22*
	P-value	0.26^#^	< 0.01^#^	
	Mean differences	− 15.01 ± 84.28	− 49.96 ± 71.02	0.04*
Fat (g/day)	Baseline	63.66 ± 20.75	70.79 ± 22.19	0.14*
	8th week	61.60 ± 18.18	58.86 ± 10.76	0.41*
	P-value	0.55^#^	< 0.01^#^	
	Mean differences	− 2.06 ± 21.69	− 11.93 ± 21.29	0.04*
Fiber (g/day)	Baseline	12.04 ± 4.27	14.09 ± 6.60	0.10*
	8th week	13.43 ± 10.18	15.96 ± 20.64	0.48*
	P-value	0.41^#^	0.59^#^	
	Mean differences	1.38 ± 10.56	1.87 ± 21.94	0.90*
Physical Activity (MET.min/week)	Baseline	437.51 ± 86.34	302.23 ± 69.43	0.22*
	8th week	475.32 ± 84.28	322.51 ± 57.25	0.13*
	P-value	0.63^#^	0.75^#^	
	Mean differences	37.81 ± 497.81	20.27 ± 401.96	0.86*
Weight (kg)	Baseline	80.31 ± 13.52	85.02.95 ± 12.99	0.11*
	8th week	79.49 ± 13.47	84.49 ± 13.11	0.09*
	P-value	0.01^#^	0.02^#^	
	Mean differences	− 0.82 ± 2.06	− 0.53 ± 1.39	0.13^†^
Height (cm)	Baseline	164.10 ± 10.04	168.95 ± 9.65	0.03*
	8th week	-	-	-
	P-value	-	-	
	Mean differences	-	-	-
BMI (kg/m^2^)	Baseline	29.71 ± 3.07	29.72 ± 3.50	0.98*
	8th week	29.40 ± 3.08	29.53 ± 3.55	0.86*
	P-value	0.01^#^	0.01^#^	
	Mean differences	− 0.30 ± 0.75	− 0.19 ± 0.47	0.11^†^
Waist circumference (cm)	Baseline	100.80 ± 8.27	100.55 ± 7.28	0.88*
	8th week	100.15 ± 8.20	99.47 ± 7.39	0.70*
	P-value	0.01^#^	< 0.01^#^	
	Mean differences	− 0.65 ± 1.67	− 1.07 ± 1.45	0.58^†^

Data are reported as mean ± SD, except for pre and post for physical activity (mean ± standard error (SE))

* Independent Sample t-test

^#^ Paired t-test

^†^ ANCOVA adjusted for changes in energy, carbohydrate, and fat intake

P-value less than 0.05 considered significant

Table 3 displays the BDI-II-Persian scores and depression status of the participants at baseline and after 8 weeks of intervention. Between-group analysis for the BDI-II-Persian score did not show any statistical differences at baseline or after 8 weeks of intervention (P > 0.05). While within-group analysis for the Diet group did not show a significant reduction in depression score (P = 0.10), it was significantly reduced in the Diet + Kefir group (mean ± SD for a baseline: 15.35 ± 10.44 and 8th week: 13.05 ± 9.30, P = 0.02). Although the BDI-II-Persian score was reduced in both groups, between-group comparison was not significant.

**Table 3:** BDI-II-Persian score and depression status of the participants at the baseline and 8th week (n = 80)

		Diet (n = 40)	Diet + Kefir (n = 40)	P-value
Depression score Mean ± SD	Baseline	17.52 ± 10.41	15.35 ± 10.44	0.35^*^
	8th week	16.15 ± 9.63	13.05 ± 9.30	0.14^*^
	P-value	0.10^#^	0.02^#^	
	Mean differences	− 1.37 ± 5.22	− 2.30 ± 6.16	0.59^†^
Depression Status				
Baseline N (%)	No or minimal	13 (32.5)	14 (35.0)	0.33^‡^
	Mild	7 (17.5)	12 (30.0)	
	Moderate	16 (40.0)	9 (22.5)	
	Severe	4 (10.0)	5 (12.5)	
8th week N (%)	No or minimal	13 (32.5)	18 (45.0)	0.71^‡^
	Mild	10 (25.0)	8 (20.0)	
	Moderate	14 (35.0)	12 (30.0)	
	Severe	3 (7.5)	2 (5.0)	

Data are reported as mean ± SD for BDI-II-Persian score and number (%) for depression status

* Independent Sample t-test

^#^ Paired t-test

^†^ ANCOVA adjusted for changes in energy, carbohydrate, and fat intake

^‡^ Chi-square test

P-value less than 0.05 considered significant

## Discussion

In our study, depression score as a secondary outcome of a randomized, single-blinded, controlled clinical trial was assessed after milk kefir consumption in NAFLD patients. Our results indicated that depression score was reduced after 8 weeks of following the diet and milk kefir consumption, but the reduction was not significantly different compared to a low-calorie diet.

Our study did not show any beneficial effects of milk kefir drink on depression scores. In contrast to our findings, a study done by Ozcan et al. [30] reported the positive effect of 500 cc kefir intervention for 30 days in postmenopausal women on Beck’s depression score, sleep disorders, and quality of life. In the study conducted by Ozcan et al. [30], post-menopausal female participants were included. The observed discrepancies could be due to the population and regulating hormonal status by kefir drinks in the above-mentioned study [30].

Nikolova and colleagues [31] conducted a meta-analysis which resulted in the beneficial effect of probiotics on depression. In addition, in the study of Akkashe et al. [32], probiotic supplementation (containing Bifidobacterium bifidio, Lactobacillus acidophilus, and Lactobacillus caseai) in patients with severe depressive disorder led to a decrease in Beck’s depression score. The source of discrepancies with our study can be related to the difference between the enrolled populations in the study. NAFLD patients suffer from dysbiosis [33], which can be a cause of both NAFLD and depression.

A regulated immune response and consequently modulated brain process can be due to a balanced intestinal microbial community which ultimately leads to improved mental state, mood, and behavior [34]. NAFLD patients show degrees of gut inflammation [35]. Thus, various pro-inflammatory cytokines are produced and transferred to the brain by systemic blood circulation. By passing the blood-brain barrier, these molecules can affect states of behavior. Tumor necrosis factor α (TNF-α) and Interleukin 6 (IL-6) can attach to their receptors and trigger cerebral Nuclear factor kappa B (NF-kB) signaling, which can upregulate the production of secondary cytokines, and finally induce depression symptoms. In addition, a higher risk of depression has been linked to higher levels of Interferon gamma (IFN-γ), Interleukin 2 (IL-2), TNF-α as pro-inflammatory cytokines, and inflammatory markers as C-reactive protein (CRP) [34].

Milk was the main ingredient of our intervention. Milk has a dual effect on depressive symptoms. Thus, skimmed milk reduces these symptoms, but whole milk increases them [19]. Milk is a good source of essential amino acids (such as tryptophan), minerals including calcium, magnesium, and zinc, and vitamins including vitamin B2 [19]. In order to produce serotonin, which can lower depression [36], tryptophan is needed as a precursor [37], and calcium is an activator for tryptophan hydrolase [38]. Magnesium and zinc with their anti-inflammatory properties can down-regulate CRP, and consequently can reduce depression [19]. Moreover, milk can provide the required magnesium to prevent magnesium deficiency which affects hypothalamic-pituitary-adrenal (HPA) dysregulation and mood changes [39]. Vitamin B2 with its role in lowering homocysteine levels by transsulfuration and remethylation pathways can lead to lower inflammation [40]. Thus, as depression is linked to inflammation, HPA dysfunction, and lower levels of serotonin, milk consumption can reduce depression symptoms [19].

Kefir drinks are also a good source for tryptophan which can modulate serotonin metabolism [41]. Thus, as depression changes the neuroplasticity, the upregulated serotonin might develop neuroplasticity [42]. On the other hand, kefir is capable of protecting the neurons from being degraded by its anti-inflammatory properties [41]. Also, the activation of receptors for learning and memory by kefir can increase cognitive improvement and finally affects depression [31]. Other pathways for depression alleviation by kefir is due to its positive effects on the gut-brain axis [43]. This pathway consists of the gamma aminobutyricacid (GABA) production after converting 2-oxoglutarate to glutamate by *Lactobacillus reuteri* [43]. Thus, the non-significant result could be due to reporting the changes in depression as a secondary outcome, while the sample size was calculated based on the changes in AST. Further investigations considering psychological profile as the principal outcome are needed to shed light on the possible effect of Kefir.

The present study was the first to assess the effect of milk kefir drink on depression in NAFLD patients. In addition, assessing the physical activity and 3-day food recalls enabled us to adjust the confounding effect of physical activity and diet. However, there were several limitations. First, as this study was an analysis of the secondary outcome, the sample size was not calculated in regard to the investigation of depression. Second, as NAFLD patients face different metabolic dysregulations such as abnormal lipid and glucose metabolism, altered gut microbiota, and dysbiosis, in the baseline, these may have a synergic effect. Moreover, for identification of individuals with NAFLD, sonography result was used, while the Fibroscan test, as a noninvasive technique, has higher accuracy than sonography in identification of NAFLD. Thus, the results of this study cannot be generalized to depressed individuals or any NAFLD patients with depression. However, it shed light on this aspect of NAFLD, and further investigations are suggested with adequate sample size, considering different abnormalities in populations, and assessment of gut microbiota population in participants.

## Conclusion

Milk kefir drink consumption for 8 weeks may not reduce depression symptoms in NAFLD patients. Further randomized controlled clinical trials with longer durations and sufficient sample size are suggested to clarify the possible effect.

## Data Availability

The datasets used and/or analyzed during the current study are available from the corresponding author on reasonable request. To access the dataset, please contact Dr. Najmeh Hejazi via email (najmehhejazi@gmail.com).

## Abbreviations

ALT: Alanine transaminase
ANCOVA: Analysis of covariance
AST: Aspartate transaminase
BDI-II-Persian: Beck Depression Inventory-II-Persian
BMI: Body mass index
CRP: C-reactive protein
DASH: Dietary Approaches to Stop Hypertension
GABA: Gamma aminobutyricacid
HPA: Hypothalamic-pituitary-adrenal
IFN-γ: Interferon gamma
IL-2: Interleukin 2
IL-6: Interleukin 6
IPAQ: International Physical Activity Questionnaire
IRCT: Iranian Registry of Clinical Trials
IU: International unit
MIND: the Mediterranean-DASH Intervention for Neurodegenerative Delay
NAFLD: Non-alcoholic fatty liver disease
NF-kB: Nuclear factor kappa B
SD: Standard deviation
SUMS: Shiraz University of Medical Sciences
T2DM: Type 2 Diabetes Mellitus
TNF-α: Tumor necrosis factor alpha
